# Involvement of folate and vitamin B12 deficiency in patients with normocytic anemia

**DOI:** 10.20407/fmj.2022-016

**Published:** 2022-10-28

**Authors:** Tatsuya Bando, Masutaka Tokuda, Itsuro Katsuda, Nobuhiko Emi, Akihiro Tomita

**Affiliations:** 1 Department of Hematology, Fujita Health University, School of Medicine, Toyoake, Aichi, Japan; 2 Department of Pharmacy, Tokai Central Hospital, Kakamigahara, Gifu, Japan; 3 Department of Clinical Hematology, Fujita Health University, Graduate School of Health Sciences, Toyoake, Aichi, Japan; 4 Department of Internal Medicine, Toyohashi Medical Center, Toyohashi, Aichi, Japan

**Keywords:** Normocytic anemia, Folate, Vitamin B12, Deficiency

## Abstract

**Objectives:**

Deficiencies in folate (FA) and vitamin B12 (VB12) are causes of macrocytic anemia. However, in clinical practice, FA and/or VB12 deficiency can occur in patients with normocytic anemia. This study aimed to determine the prevalence of FA/VB12 deficiency in patients with normocytic anemia and the importance of vitamin replacement therapy in these patients.

**Methods:**

We retrospectively reviewed electronic medical record information of patients whose hemoglobin and serum FA/VB12 concentrations were measured at the Department of Hematology (N=1,388) and other departments (N=1,421) of Fujita Health University Hospital.

**Results:**

In the Hematology Department, 530 (38%) patients showed normocytic anemia. Of these, 49 (9.2%) had FA/VB12 deficiency. Twenty of 49 (41%) patients had some hematological malignancies and 27 (55%) had benign hematological disorders. Of the nine patients who received vitamin replacement therapy, one showed a partial improvement in the hemoglobin concentration of ≥1 g/dL.

**Conclusions:**

In the clinical setting, the measurement of FA/VB12 concentrations in patients with normocytic anemia may be useful. Replacement therapy may be a treatment option to consider in patients with low FA/VB12 concentrations. However, physicians need to pay attention to the presence of background diseases, and the mechanisms of this situation require further investigation.

## Introduction

The prevalence of anemia in Japan is estimated to be 15–29/100,000 people.^1^ Additionally, anemia is still one of the most important hematological diseases, even in the era of the development of targeted therapies for some hematological disorders. An analysis of participants in the Malmö Diet and Cancer study, which was a 10-year, prospective, case–control study of more than 50,000 men and women aged 45–64 years in Sweden, reported that anemia was significantly associated with increased mortality.^2^ Anemia is also an adverse prognostic factor in patients with heart failure.^3^ In addition to patients who visit the hospital with the chief complaint of anemia, anemia may also be present in patients who are treated for other diseases. In such cases in the clinical setting, the cause of the anemia is not adequately investigated. Because anemia affects the patient’s general condition, an active search for the cause of anemia and therapeutic intervention in patients with anemia are considered important for the treatment of other diseases.

The World Health Organization defines anemia as a hemoglobin (Hb) concentration of <13.0 g/dL for adult men and <12.0 g/dL for non-pregnant adult women, although slightly lower values can be found in older adults.^4^ Anemia is classified into the three types of microcytic, normocytic, and macrocytic, according to the mean corpuscular volume (MCV), which is calculated from the red blood cell count and the hematocrit value.^5^

Microcytic anemia includes iron deficiency anemia, congenital diseases, such as thalassemia, and sideroblastic anemia caused by mutations such as *SF3B1*.^6^ Normocytic anemia is often clinically difficult to differentiate from other types of anemia due to a variety of causes, such as aplastic anemia (AA), hemolytic anemia, chronic renal failure, chronic inflammatory diseases, and early stages of microcytic/macrocytic anemia or a combination of microcytic and macrocytic anemia. Megaloblastic anemia (MBA) is the most common disease of macrocytic anemia caused by folate (FA) or vitamin B12 (VB12) deficiency. MBA is easy to distinguish clinically because the MCV is generally markedly elevated. However, in the clinical setting, screening tests in some patients with normocytic anemia show decreased serum FA/VB12 concentrations.

Jack *et al.*^7^ showed that approximately 10% of older people aged older than 65 years in the USA have anemia, and one third of them have nutritional problems. FA is one of the nutrients classified as a B vitamin, and is a coenzyme involved in amino acid and nucleic acid synthesis. FA and VB12 deficiencies are associated with impaired DNA synthesis and impaired erythropoiesis, resulting in megaloblastic anemia.^8^ Because FA and VB12 deficiencies are closely related to impaired nutrient intake, they have been investigated in relation to low nutrient intake and deficiency.

The recommended daily intake of FA is approximately 240 μg. FA deficiency can be caused by an inadequate intake, impaired absorption due to inflammatory bowel disease, increased requirement due to pregnancy and lactation, and medication. On the basis of this background, attempts are currently being made to fortify folic acid intake in food.^9^ According to a report by the National Health and Nutrition Examination Surveys in the United States, serum FA concentrations tend to be lower in men and women aged between 12 and 59 years than in other age groups. In women of childbearing age, this rate has been reported to be 0.3%.^10^

According to the Institute of Medicine Food and Nutrition Board, a daily intake of 2.4 μg of VB12 is recommended,^11^ and active intake of VB12 through supplements is recommended, especially for those older than 51 years. VB12 deficiency is caused by an inadequate intake and absorption problems due to anti-intrinsic factor antibodies and internal factor deficiency after gastrectomy. The percentage of VB12 deficiency also increases in older people, from 6% in those younger than 60 years to approximately 20% in those older than 60 years.^12^

These findings suggest that the population with FA and VB12 deficiencies is larger than that actually diagnosed with MBA. These deficiencies are often present in patients with normocytic anemia in clinical practice.^13^ Vitamin replacement therapy may contribute to the improvement of anemia in such cases. On the basis of these findings, we performed a retrospective study to determine the prevalence of FA and VB12 deficiency in patients with normocytic anemia. We also investigated whether vitamin replacement therapy improves anemia in these patients and the importance of vitamin replacement therapy for such patients.

## Materials and Methods

### Patients

**Figure 1:**
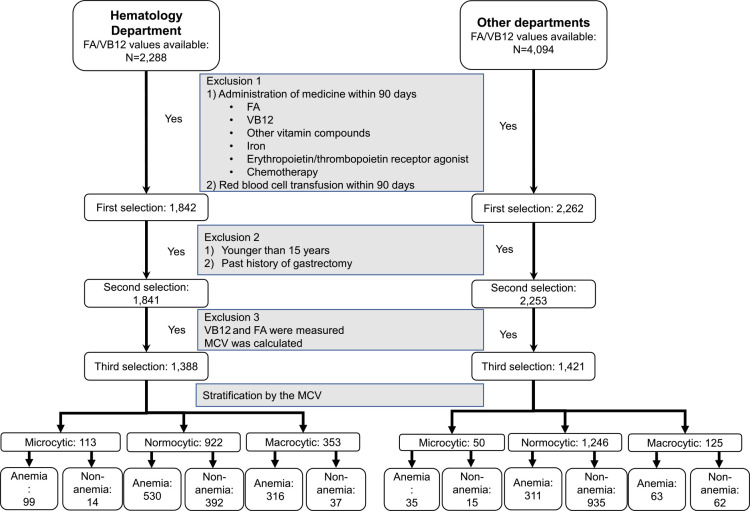
Flow chart of patients with FA and/or VB12 deficiency in the Hematology Department and other departments Patients with serum FA/VB12 data measured in the Hematology Department and other departments were selected retrospectively from electronic medical record data. Patients were selected using the eligibility criteria as indicated in the figure, and further stratification by Hb concentrations and the MCV was performed.

A total of 2,288 patients aged 14 years and older whose serum FA or VB12 concentrations were measured in the Department of Hematology, Fujita Health University Hospital, from 1 January 2004 to 30 June 2020 were included. We also included 2,781 patients aged 14 years and older whose serum FA or VB12 were measured in other departments between 1 August 2016 and 31 July 2017 (Figure 1). Patients who had received FA/VB12 supplementation within the past 90 days from the date of the first FA/VB12 measurement, those who had received iron, cytokines, antineoplastic agents, or blood products, and those who had undergone gastrectomy were excluded. This research was conducted ethically in accordance with the World Medical Association Declaration of Helsinki. This study was approved by the ethical review board of Fujita Health University School of Medicine (HM21-268).

### Definitions of anemia and FA/VB12 and iron deficiency

In accordance with the World Health Organization criteria, anemia was defined as a Hb concentration <13 g/dL for men and <12 g/dL for women,^4^ and <11 g/dL for those over 75 years of age, regardless of gender. Microcytic (MCV <80 fL), normocytic (MCV of 80–100 fL), and macrocytic (MCV >100 fL) anemia were defined on the basis of the MCV.^5^ The lower limit of the normal range of FA was set at 4 ng/dL, and its deficiency was set at <2 ng/dL. The lower limit of the normal range of VB12 was set at 300 pg/mL, and its deficiency was set at <200 pg/mL. Serum iron concentrations <40 μg/dL and ferritin concentrations <12 ng/mL were considered deficient. In cases where data were available, information on serum zinc concentrations was also collected.

### Data extraction method from electronic medical records

Patient and laboratory information was extracted from the electronic medical record system using CLISTA!SEARCH (Medical Engineering Institute, Inc., Tsu, Japan).

### Longitudinal analysis of FA and VB12 concentrations in FA/VB12-deficient patients

The effectiveness of vitamin replacement therapy in patients with normocytic anemia and FA/BV12 deficiency for 12 months after starting the medication was analyzed. Patients who had not been observed over time for more than 3 months were excluded.

### Statistical analysis

Statistical analysis was performed using EZR ver1.40 (Jichi Medical University, Saitama, Japan).^14^ The Smirnov–Grubbs rejection test was performed for outlier testing and exclusion, followed by the Mann–Whitney U test. Significance was set at *p*<0.05.

## Results

### Stratification of patients by Hb and MCV values

A total of 2,288 patients visited the Hematology Department during the analysis period and had their FA/VB12 concentrations measured. Finally, 1,388 (61%) patients were included in the analysis using the above-mentioned exclusion criteria (Figure 1). These patients were stratified into six groups according to Hb and MCV concentrations. Ninety-nine (7%) patients had microcytic anemia, 530 (38%) had normocytic anemia, and 316 (23%) had macrocytic anemia. Sixty-eight percent of patients in the Hematology Department who had their FA/VB12 concentrations measured had some form of anemia. The median age in the normocytic anemia group (N=530) and that in the normocytic non-anemia group (N=392) was 65 years and 63 years, respectively, with no significant difference between the groups (Table 1). In the normocytic anemic group, concentrations of total protein (*p*<0.001), albumin (*p*<0.001), total cholesterol (*p*<0.001), total iron binding capacity (TIBC) (*p*<0.001), unsaturated iron binding capacity (UIBC) (*p*<0.001), and the albumin/globulin ratio (*p*<0.001) were significantly lower than those in the normocytic non-anemic group (Table 1). These findings suggested that nutritional disorders may be present in some patients with normocytic anemia.

Among patients in other departments, FA or VB12 measurement was performed in 4,094 patients, and 1,421 (35%) patients were included in the analysis after exclusions. When these patients were stratified by Hb and MCV, 35 (2%) had microcytic anemia, 311 (22%) had normocytic anemia, and 63 (4%) had macrocytic anemia. Of the patients whose FA/VB12 concentrations were measured in other departments, 29% had anemia, which tended to be less than that in patients in the Hematology Department. The median age was 70 years and 66 years in the normocytic anemia group (N=311) and the normocytic non-anemia group (N=935), respectively. Patients in the anemia group were significantly older than those in the non-anemia group (*p*<0.001) (Supplementary Table S1).

### Relationship between MCV and serum FA/VB12 concentrations in patients in the Hematology Department, and FA/VB12 deficiency in patients with normocytic anemia

**Figure 2:**
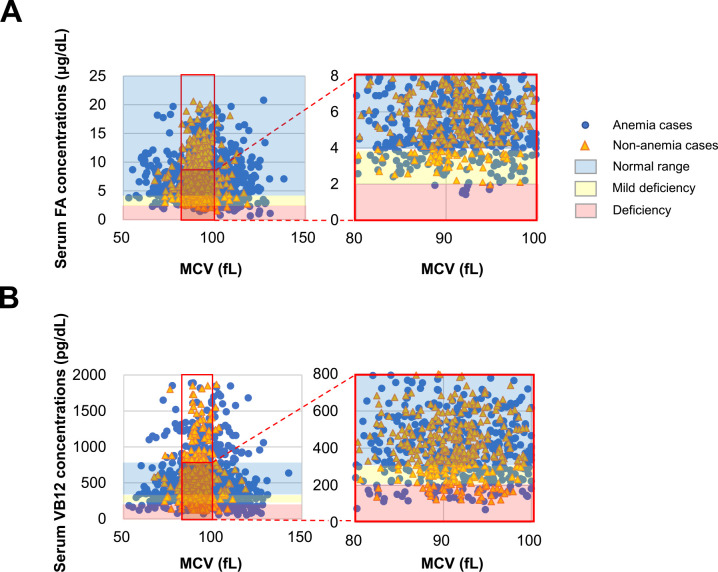
Correlations of MCV with serum FA and VB12 concentrations in anemic and non-anemic patients in the Hematology Department A) Serum FA and MCV values were evaluated in the anemia and non-anemia groups. FA deficiency (<2.0 ng/dL) and low FA concentrations (>2.0, <3.0 ng/dL) are indicated in red and yellow areas, respectively. B) Serum VB12 and MCV concentrations in the anemia and non-anemia groups were evaluated. VB12 deficiency (<200 pg/dL) and low VB12 concentrations (>200, <300 pg/dL) are indicated in red and yellow areas, respectively.

Among patients in the Hematology Department, 18 (2.7%) in the anemia group and 2 (0.5%) in the non-anemia group were FA-deficient (<2.0 mg/dL). Additionally, 165 (25%) patients in the normocytic anemia group and 60 (13.5%) patients in the normocytic non-anemia group had low FA concentrations (≥2.0, <4.0 mg/dL) (Figure 2A). VB12 deficiency (<200 pg/dL) was observed in 98 (14.8%) patients in the normocytic anemia group and 38 (8.6%) patients in the normocytic non-anemia group. Additionally, low VB12 concentrations (≥200, <300 pg/dL) were observed in 158 (24%) patients in the normocytic anemia group and in 51 (11.5%) patients in the normocytic non-anemia group (Figure 2B). Therefore, low FA/VB12 concentrations were present not only in the normocytic anemic group, but also in the normocytic non-anemic group.

**Figure 3:**
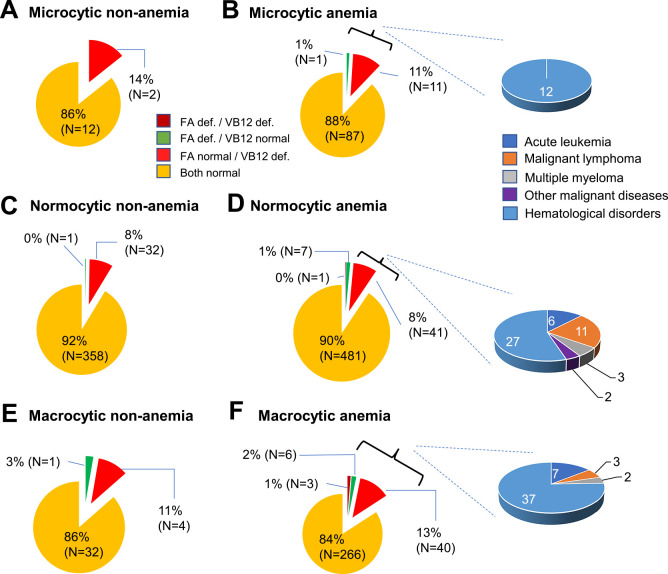
FA and/or VB12 deficiency in patients with or without anemia in the Hematology Department The numbers of patients with FA and/or VB12 deficiency are shown in each group classified by the MCV and the anemic status. A) Microcytic non-anemia, B) microcytic anemia, C) normocytic non-anemia, D) normocytic anemia, E) macrocytic non-anemia, and F) macrocytic anemia groups are shown. def.: deficiency.

A detailed breakdown of the FA/VB12 deficiency status in the microcytic, normocytic, and macrocytic non-anemia and anemia groups, and the underlying diseases in vitamin-deficient patients are shown in Figure 3. Patients with normocytic anemia included cases of vitamin deficiency as follows: 1 FA/VB12-deficient, 7 (1%) FA-deficient, and 41 (8%) VB12-deficient patients. When we compared vitamin concentrations between the groups (Table 1), mean FA concentrations were significantly lower in the normocytic anemia group than in the normocytic non-anemia group (*p*<0.001). However, there was no significant difference in VB12 concentrations between the groups. The white blood cell count was significantly lower in the normocytic anemia group than in the normocytic non-anemia group (*p*<0.001). These findings suggested that some type of hematopoietic failure may have been partly responsible for normocytic anemia in patients in the Hematology Department.

### Investigation of the underlying diseases in patients with FA/VB12 deficiency in the Hematology Department

The primary diseases in normocytic anemia with FA and/or VB12 deficiency (N=49) were as follows: malignant neoplastic diseases in 22 (45%) patients, acute leukemia in 6 (12%), malignant lymphoma in 11 (22%), multiple myeloma in 3 (6%), and a solid tumor in 2 (4%); and other hematological disorders in 27 (55%). The prevalence of malignant neoplastic diseases in patients with microcytic and macrocytic anemia with vitamin deficiency was 0% and 24%, respectively. These findings suggested that involvement of hematological malignancies may be more important in patients with normocytic anemia with FA and/or VB12 deficiency than in patients with microcytic/macrocytic anemia. Other hematological disorders may also be important.

### Comparison of serum zinc, iron, and ferritin values in the normocytic anemia and non-anemia groups in the Hematology Department

Serum zinc concentrations were significantly lower in the normocytic anemia group than in the normocytic non-anemia group (*p*<0.001, Table 1). Serum iron concentrations in the two groups were analyzed separately by sex and in older people. We found that iron concentrations were lower in men and women in the normocytic anemia group than in the normocytic non-anemic group (*p*=0.01 and* p*=0.03, respectively). Interestingly, there was no significant difference in ferritin concentrations between the groups, regardless of sex or age. These findings suggested that, although iron tended to be low in patients with normocytic anemia, the amount of stored iron had not decreased. Therefore, the effect on anemia may be small.

### Iron deficiency status in patients with normocytic anemia showing FA and/or VB12 deficiency

In the clinical setting, the coexistence of iron deficiency may sometimes be considered if FA and/or VB12 deficiency is confirmed in normocytic anemia. However, the actual situation is still unclear. Therefore, whether iron deficiency (iron concentrations <40 mg/dL, ferritin concentrations <12 ng/mL) coexists in patients with normocytic anemia showing FA and/or VB12 deficiency was examined (Table 2). In patients with normocytic anemia (N=300), iron deficiency was observed in 19 (6%), of whom only 2 showed VB12 deficiency and no FA deficiency. This result indicated that the coexistence of iron deficiency in patients who had normocytic anemia, despite FA and/or VB12 deficiency, was rare.

We also analyzed the association between serum zinc concentrations and FA and VB12 concentrations. Although the number of patients who had zinc concentrations measured was limited (Table 1), a weak correlation was found between zinc concentrations and FA or VB12 deficiency (Supplementary Figure S1).

### Effect of FA and VB12 replacement therapy in patients with normocytic anemia and FA and/or VB12 deficiency

**Figure 4:**
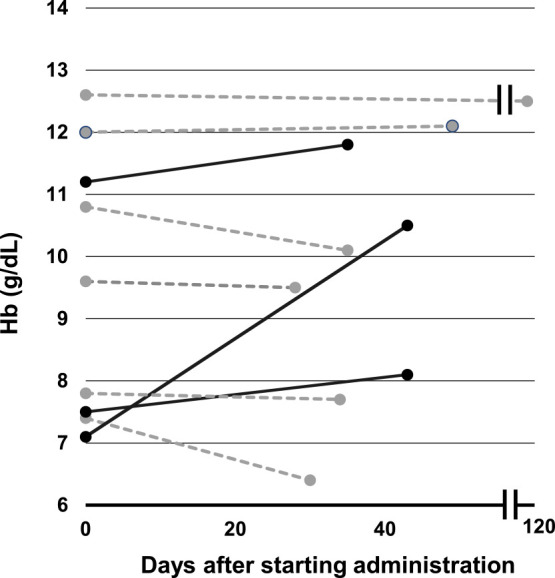
Effectiveness of vitamin replacement therapy in patients with normocytic anemia and FA/VB12 deficiency in the Hematology Department The effectiveness of FA or VB12 replacement therapy was evaluated retrospectively. The changes in Hb concentrations after the start of replacement therapy are shown. Each line indicates an individual patient. Solid lines indicate patients showing elevated Hb concentrations, and the dotted line indicates non-responders.

The effect of vitamin replacement therapy on patients with normocytic anemia showing FA and/or VB12 deficiency has not been clarified. Among the patients with normocytic anemia in the Hematology Department who showed FA and/or VB12 deficiency, the effect of replacement therapy was retrospectively examined in nine patients (Figure 4). An improvement in anemia was found in one (11%) patient with a ≥1 g/dL elevation in Hb concentrations, and a minor elevation of <1 g/dL was found in two patients. Six (67%) patients did not show any effect. The underlying diseases in the responder group were myelodysplastic syndromes (MDS) in one, aplastic anemia (AA) in one, and disseminated intravascular coagulation in one patient. The non-responder group included three patients with AA and one patient with MDS. The effects of replacement therapy on patients with normocytic anemia and FA and/or VB12 deficiency were limited, which suggested that there was diversity in the pathogenesis.

### Status of anemia in patients in other departments, and the relationships between MCV, FA concentrations, and VB12 concentrations

We found that patients in other departments (N=1,421) tended to have less dispersion of MCV values than those who visited the Hematology Department (interquartile range: 87.1–96.9 vs. 88.1–103.4) (Supplementary Figures S2 and S3). FA deficiency was found in 9 (2.2%) patients, and VB12 deficiency was found in 36 (8.8%) patients in the normocytic anemia group (N=409) (Supplementary Figure S2). In the analysis of the anemia group classified by the MCV (Supplementary Figure S4), FA deficiency was found in 4 (1.3%) patients, and VB12 deficiency was found in 30 (9.6%) patients in the normocytic anemia group (N=311). This finding indicated that vitamin deficiency was present in approximately 10% of patients with normocytic anemia.

The normocytic group was divided into the anemia group and the non-anemia group, and the examination values were compared (Supplementary Table S1). Zinc concentrations were significantly lower in the anemia group than in the non-anemia group (*p*<0.001). Additionally, iron concentrations were significantly lower in the anemia group than in the non-anemia group, regardless of whether patients were men, women, or older (*p*<0.001, *p*<0.01, and *p*<0.001, respectively).

However, ferritin concentrations appeared to be lower in the anemia group than in the non-anemia group, but this was not significant. No significant difference in serum FA or VB12 concentrations was found between the anemia and non-anemia groups. None of the patients in other departments underwent replacement therapy in those with normocytic anemia with FA/VB12 deficiency (N=34). When the background diseases in these 34 patients were examined, they were heart failure in 8 (23%), myocardial infarction in 4 (12%), and deep vein thrombosis in 2 (5.8%) patients.

These findings suggested that there were a certain number of patients with normocytic anemia who had a background of vitamin deficiency, even in patients in other departments. No information was obtained about the efficacy of vitamin replacement therapy in this limited retrospective cohort.

## Discussion

In this study, a survey of the vitamin deficiency status in patients with normocytic anemia was conducted, and the importance of vitamin replacement therapy in these patients was retrospectively investigated. We found that approximately 10% of patients, with or without anemia, had low FA or VB12 concentrations, which is more than expected. This study also showed that an improvement of anemia was observed only in a limited number of patients in whom vitamin replacement therapy was performed for patients with vitamin deficiency and normocytic anemia. This finding suggests that vitamin replacement therapy is a candidate for the treatment strategy in such patients, but the cause of the anemia should be further investigated considering the possibility of the effects of other diseases.

This study showed that the proportion of patients with malignant diseases, such as hematological malignancies as the primary diseases, was higher in the normocytic anemia group with FA/VB12 deficiency than in the microcytic and macrocytic anemia groups with vitamin deficiency. One of the reasons for lacking the macrocytic phenotype, despite FA/VB12 deficiency, may due to secondary anemia caused by the presence of malignant disease or chronic inflammatory disease.^15^ When there is anemia in wasting diseases, microcytic anemia is common, but because of the concomitant vitamin deficiency, the MCV level may not be typical in size and may remain in the range of normocytic anemia. More detailed analyses, such as the tumor status and nutritional status, are required in individual cases.

Hematopoietic disorders, such as AA and MDS, were also primary diseases in patients with normocytic anemia with vitamin deficiency. AA was characterized by pancytopenia due to dysfunction of hematopoietic stem cells, mostly owing to autoimmune mechanisms, but there might have been cases of vitamin deficiency in the background of hematopoietic disorders. In such cases, vitamin deficiency might not show typical MBA. Similarly, in patients with hematopoietic disorders^16^ due to genetic abnormalities such as MDS, the presence of vitamin deficiency might not result in marked macrocytic anemia. This possibility might be an explanation for patients in whom replacement therapy for vitamin deficiency did not provide a clear effect. To clarify these possibilities, a rigorous diagnosis of background diseases in patients with normocytic anemia and vitamin deficiency is required. Additionally, the effectiveness of vitamin replacement therapy in such patients needs to be prospectively investigated.

A recent report that examined the accumulation of genetic abnormalities in peripheral blood in healthy participants showed that the prevalence of mosaic chromosomes increased from the age of 50 years.^17^ Additionally, clonal hematopoiesis with somatic mutations was observed in approximately 10% of people aged 60 years and older.^18^ With the accumulation of these genetic abnormalities with aging, mild hematopoietic disorders, such as cloning hematopoiesis of indeterminate potential, and cloning cytopenia of undetermined significance are thought to appear.^19,20^ The median age in this cohort was older than 60 years, and even if not diagnosed as a blood disorder, the possibility of the existence of mild hematopoietic disorders when these genetic abnormalities are present cannot be excluded. Examination of genetic abnormalities in patients with vitamin deficiency with normocytic anemia might be useful not only in detecting the underlying pathology, but also in developing more appropriate treatments for anemia for specific cases.

In the clinical setting, iron deficiency anemia may be associated with the presence of normocytic anemia, despite being in a vitamin deficiency state. However, in the present study cohort, such patients were extremely rare. When administering iron to such patients, measuring the serum ferritin concentration before iron replacement therapy is important.

In the present study, a similar analysis of patients in departments other than the Hematology Department was also conducted. We found that the degree of dispersion of the MCV in patients who visited the Hematology Department was wider than that in patients who visited other departments. This finding may reflect the fact that the background diseases in patients with anemia are different in each department. We also found that patients with normocytic anemia with vitamin deficiency (N=36) also had heart failure, cardiovascular disease, or deep vein thrombosis. The possibility that anemia due to vitamin deficiency may have contributed to the worsening of heart failure cannot be ruled out. A previous report showed that FA/VB12 deficiency caused elevated homocysteine concentrations in the blood, which can be a risk factor for thrombosis.^21^ This previous finding suggested that FA/VB12 deficiency may have been partially involved in cardiovascular disorders in this cohort. However, the existence of these diseases and the mechanism that cause normocytic anemia are still unclear. A limitation of this study is that it did not examine the use of various drugs (e.g., omeprazole, rabeprazole, lansoprazole, vonoprazan, metformin, and valproic acid) that may inhibit the absorption of vitamins and other substances. This is a clinically important point of focus, and future studies on the differences in vitamin concentrations due to oral medication in combined disease groups are warranted. Further accumulation of cases and a careful investigation of background factors, such as the effects of medication, are required.

The present retrospective study showed that there was a certain number of cases of FA and/or VB12 deficiency in patients with normocytic anemia, and partial effectiveness of replacement therapy was shown in a limited number of patients. Future studies of the efficacy of replacement therapy for patients with these characteristics are warranted. An analysis of the molecular mechanisms involved in FA and/or VB12 deficiency in anemia to improve the treatment of anemia should also be conducted.

## Figures and Tables

**Table1:** Laboratory data of patients in the Hematology Department with normocytic anemia or normocytic non-anemia

	Anemia group (N=531)		Non-anemia group (N=392)		*p*
Men, N (%)	270 (51)		217 (55)		
Age (years),	65 (16–91)		63 (16–95)		*0.09*
Hemoglobin (g/dL)	Men (N=186)	10.4 (6.3–13)	Men (N=138)	15.1 (13.1–21.5)	<0.001*
	Women (N=198)	10.0 (5.1–12)	Women (N=117)	13.4 (12.1–18.8)	<0.001*
	Older (N=49)	9.0 (5.1–11)	Older (N=24)	12.9 (11.2–18.2)	<0.001*
MCV (fL)	91.6 (80–109.9)		91.4 (80–100)		*0.47*
White blood cell (×10^3^/μL) (N=487:372)	5.2 (0.5–15.1)		6.0 (1.2–15.3)		<0.001*
Platelet (×10^4^/μL) (N=524:383)	17.5 (0.1–59.6)		18.5 (0.1–62)		*0.17*
Total protein (g/dL) (N=483:382)	6.8 (4.2–9.8)		7.0 (4.9–8.8)		<0.001*
Albumin (g/dL) (N=477:375)	3.8 (1.4–5.2)		4.2 (2.4–5.2)		<0.001*
Albumin/globulin (N=476:375)	1.25 (0.22–2.63)		1.48 (0.57–2.69)		<0.001*
Total cholesterol (pg/mL) (N=374:318)	158 (51–312)		188 (101–329)		<0.001*
Lymphocyte count (N=510:383)	1.08 (0.11–1.62)		1.36 (0.13–3.71)		<0.001*
Reticulocyte count (N=474:368)	15 (1–41)		14 (3–35)		0.03*
Folic acid (ng/mL) (N=510:380)	6.7 (1.4–19)		7.5 (2.0–20.6)		<0.001*
Vitamin B12 (pg/mL) (N=461:373)	527 (70–1890)		522 (113–1850)		*0.83*
Zinc (μg/dL) (N=148:133)	61.0 (16–99)		69.7 (37–99)		<0.001*
Iron (μg/dL) (N=519)	Men (N=126)	79.3 (8–250)	Men (N=85)	97.7 (19–247)	0.01*
	Women (N=121)	73.8 (8–283)	Women (N=73)	90.3 (19–253)	0.03*
	Older (N=29)	72.0 (13–190)	Older (N=16)	70.6 (36–186)	*0.80*
Ferritin (ng/mL) (N=378)	Men (N=110)	273 (6–908)	Men (N=75)	222 (13–743)	*0.11*
	Women (N=117)	170 (3–1019)	Women (N=68)	114 (4–848)	*0.05*
	Older (N=30)	246 (10–908)	Older (N=15)	127 (11–684)	<0.001*
TIBC (μg/dL) (N=303:198)	264 (98–526)		306 (136–483)		<0.001*
UIBC (μg/dL) (N=309:205)	190 (5–512)		218 (26–422)		<0.001*
Transferrin saturation (%) (N=304:198)	22.48 (2.27–97.79)		26.99 (6.86–90.04)		*0.06*

Values are N (%), N, or median (range). MCV: mean corpuscular volume, TIBC: total iron binding capacity, UIBC: unsaturated iron binding capacity. *The Mann-Whitney U test showed a significant difference.

**Table2:** Patients with FA and/or VB12 deficiency and iron deficiency anemia

		Iron deficiency		
		Total	VB12 deficiency	FA deficiency
Hematology Department	Microcytic anemia (N=72)	37	6	0
	Normocytic anemia (N=300)	19	2	0
	Macrocytic anemia (N=161)	0	0	0

Other departments	Microcytic anemia (N=19)	10	2	0
	Normocytic anemia (N=86)	1	0	0
	Macrocytic anemia (N=31)	0	0	0
