# Supplementary material for: Involvement of folate and vitamin B12 deficiency in patients with normocytic anemia
Source: Fujita Med J. 2022 Oct 28;9(2):134–41. doi: 10.20407/fmj.2022-016 (PMC10206897; doi:10.20407/fmj.2022-016)
Supplement: Supplementary file 1 — Supplementary Figures [file fmj-9-134-s001.pdf]

Supplementary figure legends

Supplementary Figure S1

Correlations of serum zinc concentrations with serum FA and VB12 concentrations in anemic and non-anemic patients in the Hematology Department

Correlations of serum zinc concentrations with FA **A)** concentrations and VB12 concentrations **B)**. The coefficient of determination is indicated as R^2.

Supplementary Figure S2

Correlation of the MCV with serum FA and VB12 concentrations in anemic and non-anemic patients in departments other than the Hematology Department

A) Serum FA and MCV values in the anemia and non-anemia groups were evaluated. FA deficiency (<2.0 mg/dL) and low FA concentrations (≥2.0, <4.0 mg/dL) are indicated in red and yellow areas, respectively. **B)** Serum VB12 and MCV values in the anemia and non-anemia groups were evaluated. VB12 deficiency (<200 pg/dL) and low VB12 concentrations (≥200, <300 pg/dL) are indicated in red and yellow areas, respectively.

Supplementary Figure S3

Comparison of the distribution of the MCV among anemic patients in the Hematology Department and other departments

Data of MCV in anemic patients in the Hematology Department and other departments were analyzed, and the median, range, and interquartile range

values are shown.

Supplementary Figure S4

FA and/or VB12 deficiency in patients with or without anemia in departments other than the Hematology Department

The numbers of patients with FA and/or VB12 deficiency are shown in each group classified by the MCV and the anemic status. **A)** The microcytic non-anemia, **B)** microcytic anemia, **C)** normocytic non-anemia, **D)** normocytic anemia, **E)** macrocytic non-anemia, and **F)** macrocytic anemia groups are shown. def.: deficiency.

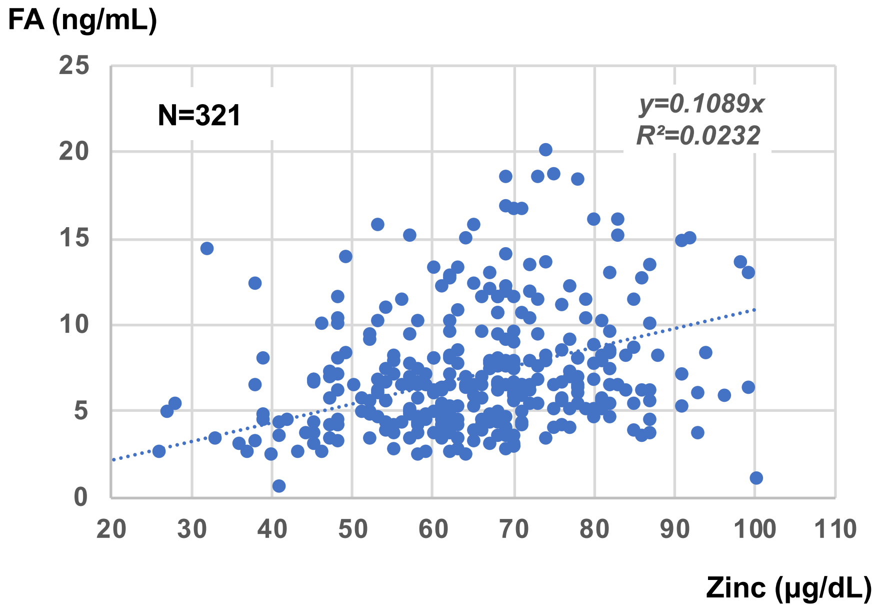

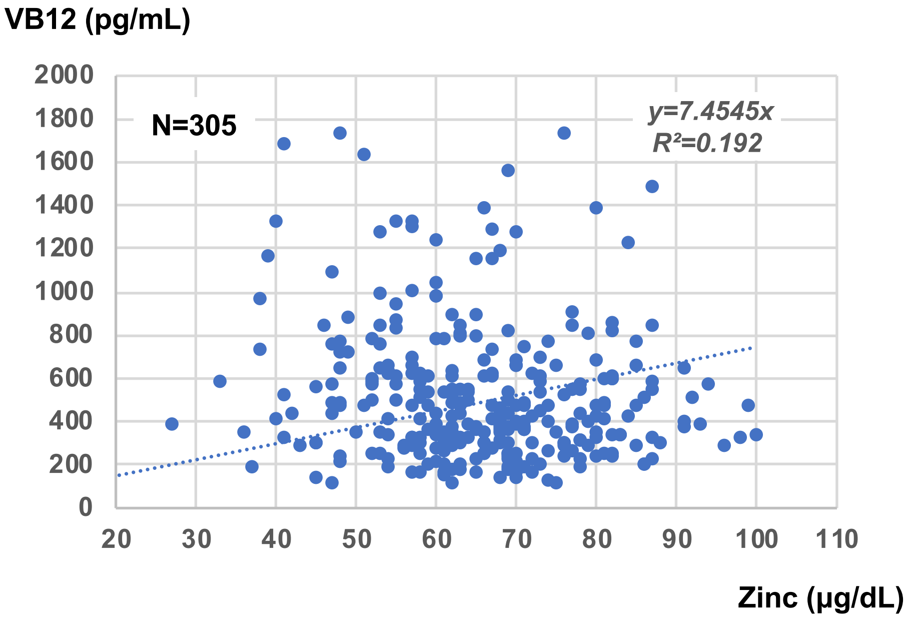

Supplementary Figure S1

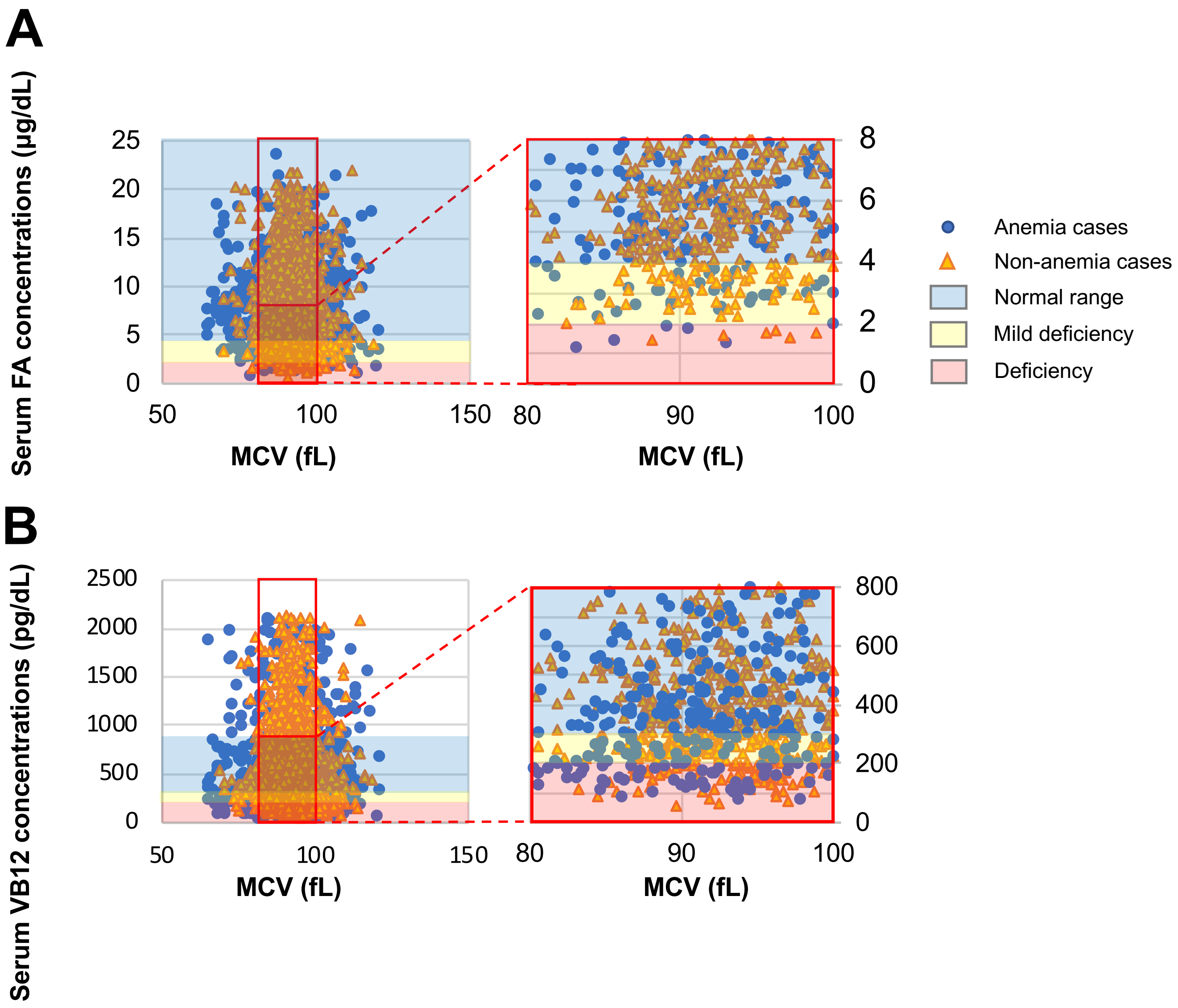

Supplementary Figure S2

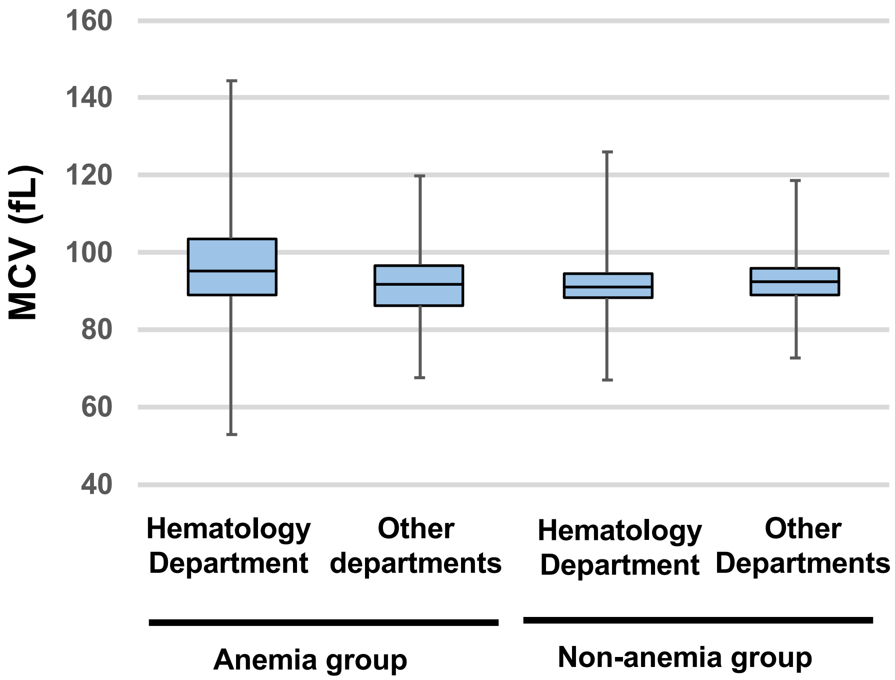

Supplementary Figure S3

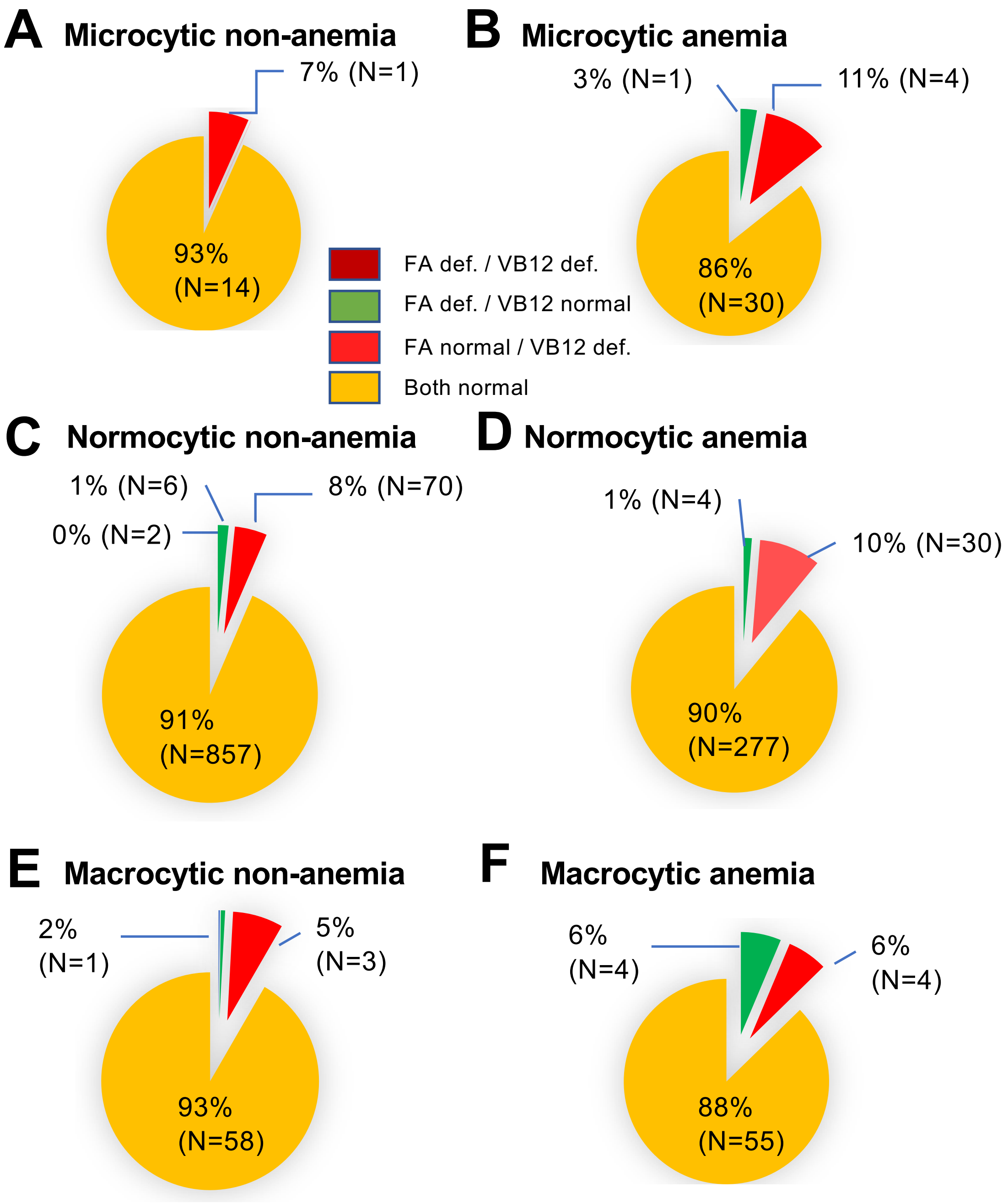

Supplementary Figure S4

Supplementary Table S1

Laboratory data of patients in other departments with normocytic anemia and normocytic non-anemia

	Anemia group (N=311)		Non-anemia group (N=935)		*p*
**Men,** N (%)	154 (50)		530 (57)		
**Age** (years), median (range)	70 (17- 97)		66 (15-94)		*<0.001**
**Hemoglobin** (g/dL), median (range)	Men (N=105)	11.7 (6.9-13)	Men (N=317)	15.0 (13.1-18.3)	*<0.001**
	Women (N=76)	10.7 (8.0-12.0)	Women (N=233)	13.7 (12.1-17.5)	*<0.001**
	Older (N=130)	9.6 (6.4-11)	Older (N=385)	13.0 (11.1-18.1)	*<0.001**
**MCV** (fL), (range) (IQR)	91.2 (80-100) (87.8-95.0)		91.8 (80-100) (88.8-94.8)		*0.05*
**White blood cell** (x10^3/μL) (N=309:928)	7.5 (0.6-18.5)		7.4 (1.3-18.3)		*0.73*
**Platelet** (x10^4/μL) N=(306:935)	22.3 (0.9-50.5)		22.2 (3.8-46.3)		*0.79*
**Folate** (ng/mL) (N=304:917)	7.9 (1.5-21.3)		7.6 (1.0-21.7)		*0.36*
**Vitamin B12** (pg/mL) (N=283:888)	537 (87-1981)		548 (59-1991)		*0.65*
**Zinc** (μg/dL) (N=31:174)	65 (34-89)		75 (43-116)		*<0.001**
**Iron** (μg/dL) (N=277)	Men (N=31)	56 (9-182)	Men (N=50)	87 (25-183)	*<0.001**
	Women (N=35)	65 (22-217)	Women (N=59)	91 (33-147)	*<0.01**
	Older (N=42)	47 (8-165)	Older (N=60)	69 (12-147)	*<0.001**
**Ferritin** (ng/mL) (N=193)	Men (N=22)	160 (14-609)	Men (N=29)	199 (11-737)	*0.43*
	Women (N=26)	134 (5-672)	Women (N=35)	110 (9-457)	*0.46*
	Older (N=38)	171 (7-703)	Older (N=43)	202 (17-744)	*0.45*

MCV: mean corpuscular volume, IQR; interquartile range. *The Mann-Whitney U test showed a significant difference.
